# Soil-Borne Bacterial Structure and Diversity Does Not Reflect Community Activity in Pampa Biome

**DOI:** 10.1371/journal.pone.0076465

**Published:** 2013-10-16

**Authors:** Manoeli Lupatini, Afnan Khalil Ahmad Suleiman, Rodrigo Josemar Seminoti Jacques, Zaida Inês Antoniolli, Eiko Eurya Kuramae, Flávio Anastácio de Oliveira Camargo, Luiz Fernando Würdig Roesch

**Affiliations:** 1 Departamento de Solos, Universidade Federal de Santa Maria, Santa Maria, Rio Grande do Sul, Brazil; 2 Department of Microbial Ecology, Netherlands Institute of Ecology (NIOO/KANW), Wageningen, The Netherlands; 3 Departamento de Solos, Universidade Federal do Rio Grande do Sul, Porto Alegre, Rio Grande do Sul, Brazil; 4 Universidade Federal do PAMPA, Campus São Gabriel, São Gabriel, Rio Grande do Sul, Brazil; Dowling College, United States of America

## Abstract

The Pampa biome is considered one of the main hotspots of the world’s biodiversity and it is estimated that half of its original vegetation was removed and converted to agricultural land and tree plantations. Although an increasing amount of knowledge is being assembled regarding the response of soil bacterial communities to land use change, to the associated plant community and to soil properties, our understanding about how these interactions affect the microbial community from the Brazilian Pampa is still poor and incomplete. In this study, we hypothesized that the same soil type from the same geographic region but under distinct land use present dissimilar soil bacterial communities. To test this hypothesis, we assessed the soil bacterial communities from four land-uses within the same soil type by 454-pyrosequencing of 16S rRNA gene and by soil microbial activity analyzes. We found that the same soil type under different land uses harbor similar (but not equal) bacterial communities and the differences were controlled by many microbial taxa. No differences regarding diversity and richness between natural areas and areas under anthropogenic disturbance were detected. However, the measures of microbial activity did not converge with the 16S rRNA data supporting the idea that the coupling between functioning and composition of bacterial communities is not necessarily correlated.

## Introduction

The Pampa biome is considered one of the main hotspots of the world’s biodiversity and is one of the priority areas for flora and fauna conservation [1], [2]. Despite its importance, it is estimated that half of its original vegetation was removed and converted to agricultural land and to large areas with exotic tree plantation [3], [4]. Although an increasing amount of knowledge is being assembled regarding the response of soil bacterial communities to land use types, the associated plant community and soil properties [5], [6], [7], [8], our understanding about how the interaction among land use and soil type affect the microbial community from the Brazilian Pampa is still poor and incomplete. It is well known that bacterial communities are the most abundant and diverse group of soil microorganisms and exert multiple important key roles on soil, such as decomposition, biogeochemical cycles and nutrient transformations and any modifications in the microbial community caused by land use change might contribute for changing the ecosystem functions and maintaining soil quality [9].

Particularly the aboveground vegetation affects the structure, size and activity of soil microbial communities through the input of different quantity and quality of litter deposition in soil [6], [10]. On the other hand, many studies have reported that soil properties and land use might be considered as keys factors affecting bacterial diversity and composition. Soil pH and texture [11], [12], [13], [14], Ca/Mg ratio, Al, Ca, Mg, K, B and P contents [15], [16] are considered the major factors. Bacterial communities can also be affected by others factors, i.e. history of land use, which was considered a stronger determinant of the composition of microbial communities than vegetation and soil properties [17], [18]. In addition, Girvan et al. [19] proposed that the soil type is the primary determinant of the bacterial community composition in arable soils, but to date, little information is available about the ecological interaction between soil type and bacterial communities.

Nevertheless, land use does not always have a significant effect on soil bacterial community. Despite major differences in soil properties and vegetation, soil microbial communities in mature grasslands and deciduous forests were similar [18], [20]. This similarity might be caused by some microbial groups that show a high degree of tolerance to changes in environmental conditions, that might result in microbial communities that are resistant or resilient to disturbances caused by land use [21]. The knowledge of how microbial diversity are influenced by soil management in the Brazilian Pampa may help us to understand the changes in carbon balance, energy flow, and greenhouse gas fluxes under these shifted areas Such knowledge is fundamental for the sustainable management of the soil ecosystem in this threatened hotspot of biodiversity.

Assuming that both land use and the soil type affect the bacterial communities, here we performed a large-scale pyrosequencing-based analysis of the 16S rRNA gene to evaluate bacterial diversity, composition and structure from the same soil type but with different land uses. Also, we analyzed the soil microbial activity through the measurements of the microbial biomass carbon and the metabolic quotient. We hypothesized that the same soil type from the same geographic region but under distinct land use presents dissimilar soil bacterial communities. In order to test our hypothesis we assessed and compare the impact of land use under the same soil type on soil bacterial communities from the Brazilian Pampa biome by sampling typical land uses found in this region. Our major goal was to obtain a detailed baseline description of the soil bacterial communities found in the Brazilian Pampa soils against which to compare changes in the soil microbiome caused by human activities.

## Materials and Methods

### Site Description, Soil Sampling and Soil Physicochemical Analysis

In order to analyze the impact caused by land use change on the bacterial community, soil samples were collected in a site with four typical land uses in the Pampa biome. This biome covers an area shared by Brazil, Argentina and Uruguay in the southern of South America and is characterized by typical vegetation of native grassland, with sparse shrub and tree formations [22]. In Brazil, this biome occupy part of Rio Grande do Sul State, has both subtropical and temperate climates with four well-characterized seasons, and was officially recognized by the Brazilian Institute of Geography and Statistics only in 2004 [23] (Figure S1). To minimize the effect of climate and soil type on microbial community at each site, samples were collected at the same day (November, 2010), in adjacent areas and under the same soil type (PALEUDULT, Soil Taxonomy), distant 500 meters apart. The samples were collected in a private land and no specific permissions were required for soil sampling. Also, our study did not involve endangered or protected species. Soil samples were collected in following land uses: NP: natural pasture (30° 00′ 38.2″ S and 54° 50′ 17.4″ W, altitude 121 m) currently used for intensive grazing of cattle, with no fertilizers input (except for the manure added by animal activity) or introduction of exotic species; NF: natural forest (30° 00′ 39.7″ S and 54° 50′ 05.6″ W, 150 m – control sample) used only for preservation of wildlife with no fertilizers inputs and no human activity or animal influence; SF: soybean field (30° 00′ 40.3″ S and 54° 50′ 13.2″ W, 137 m) cultivated under no-tillage system on oat straw, with plants in early growth stage; and AP: 9 years old acacia trees (*Acacia mearnsii* Willd.) plantation (30° 00′ 27.5″ S and 54° 50′ 10.2″ W, altitude 141 m).

Bulk soil samples were collected following the experimental design proposed by Baker et al. [24]. The samples were taken by drawing four randomly distributed 1 m^2^ squares approximately 80 m apart to each other within each land use. The distance among plots were determined according to Wallenius et al. [25], who found that most of the microbiological characteristics are independent when samples are taken 0.5 m apart. In each plot, composite samples were collected by taking sub-samples in every corner of the square. Soil samples were collected taking 5 cm diameter, 0–5 cm depth cores. Equal masses of sub-samples removed from cores were pooled and mixed to form four composite samples from each land use. All samples were packed on ice upon collection and transported to the laboratory and kept at −18°C up to the microbial DNA extraction and chemical analysis. From each composite sample, a subsample was removed, air dried and 2 mm mesh sieved and subjected to the chemical and physical analysis. The physicochemical analysis was performed according to the recommendations of the Brazilian Society of Soil Science [26]. Additionally, to illustrate soil factors of different land use, principal component analysis (PCA) was carried out in R [27].

### DNA Extraction, 16S rRNA Partial Gene Amplification and Pyrosequencing

Soil DNA was extracted with the PowerSoil® DNA Kit (MoBio, Carlsbad, CA, USA) according to the manufacturer’s instruction with the exception that 1 g rather than 0.25 g of soil was used and the final DNA extracts were eluted into 50 µL of ultrapure H_2_O rather than solution C6. DNA concentrations were determined using NanoVue™spectrophotometer (GE Healthcare) and all DNA samples were stored at −20°C. Independent PCR reactions were performed for each soil sample with the primers 27F and 338R described in Fierer et al. [28] for the amplification of approximately 311 base pairs of the V1–V2 region of the 16S rRNA gene. PCR reactions were carried out in triplicate with the GoTaq PCR core system (Promega, Madison, WI, USA). The mixtures contained 5 µl of 10X PCR buffer, 200 mM dNTPs, 100 mM of each primer, 2.5 U of *Taq* polymerase and approximately 100 ng of DNA template in a final volume of 50 µl. The PCR conditions were 94°C for 2 minutes, 30 cycles of 94°C for 45 s; 55°C for 45 s; and 72°C for 1 minutes extension; followed by 72°C for 6 minutes. The PCR products for each of the 16 samples were purified and combined in equimolar ratios with the quantitative DNA binding method (SequalPrep Kit, Invitrogen, Carlsbad, CA, USA) for DNA pool for pyrosequencing from the A-Key adaptor. The 16S rRNA gene fragments were sequenced using 454 GS FLX Titanium (Lib-L) chemistry for unidirectional sequencing of the amplicon libraries. Barcoded primers were used to multiplex the amplicon pools in order to be sequenced together and computationally separated afterward. To do this, 8-base barcodes were added to the 5′ -end of the reverse primers using the self-correcting barcode method of [29]. The primers were attached to the GS FLX Titanium Adaptor A-Key (5′-CCATCTCATCCCTGCGTGTCTCCGACTCAG -3′) and Adaptor B-Key (5′-CCTATCCCCTGTGTGCCTTGGCAGTCTCAG-3′) sequences, modified for use with GS FLX Titanium Em PCR Kits (Lib-L) and a two-base linker sequence was inserted between the 454 adapter and the 16S rRNA primers to reduce any effect the composite primer might have on PCR efficiency. All raw sequences were submitted to the NCBI Sequence Read Archive (SRA) under the study number SRP013204, experiment number SRX255448.

### Processing of Pyrosequencing Data and Taxonomic Assignments

The raw sequences obtained were processed using QIIME [30] with default parameters. Briefly, to reducing sequencing errors and their effects, the multiplexed reads were first filtered for quality and assigned to the starting soil samples. The filtering criteria included a perfect match to the sequence barcode and primer, at least 200 bp in length, no undetermined bases, and at least 60% match to a previously determined 16S rRNA gene sequence [29]. Additionally, to identify potentially chimeric sequences, the dataset were subject to the ChimeraSlayer implemented in mothur [31]. The output fasta file was used for building a table with the Operational Taxonomic Unit (OTU) abundance of each sample and the taxonomic assignments for each OTU. To do this, the sequences were clustered into OTUs based on the relatedness of the sequences (97% similarity) and a representative sequence from each OTU was selected. These representative sequences were subjected to the RDP naïve Bayesian rRNA Classifier [32], which attaches complete taxonomic information from domain to species to each sequence in the database with 80% taxonomy confidence and an e-value of 0.001. The representative set of sequences was also used to align the sequences against the greengenes 16S rRNA database [33] and to build a phylogenetic tree necessary for downstream measurements.

### Alpha and Beta Diversity Analysis

For each taxonomic level (Phylum, Class, Order, Family and Genus) and at 97% similarity cutoff Good’s coverage was calculated [34]. To compare the similarity between bacterial communities from the soil samples we estimated the diversity of each sample using alpha Phylogenetic Diversity - PD and Rényi diversity profiles. The Phylogenetic Diversity is defined and calculated as the sum of the branch-lengths of the minimal sub tree connecting the taxa in the subset [35]. This evaluation is based on a single phylogenetic tree and is sensitive to the quality of the branch length and topology. Rényi diversity profiles provide information on diversity, richness and evenness of the community. Each value of the Rényi diversity profile is based on an alpha parameter. This diversity ordering technique is preferred to ranking based on single indices because rank order may change when different indices are used [36]. The shape of the profile is an indication of the evenness, a horizontal profile indicates that all species have the same evenness and the less horizontal a profile is, the less evenly species are distributed.

The starting position at the left-hand side of the profile is an indication of the species richness (alpha = 0) and the diversity is ordered from high to low diversity profiles. Profiles that start at a higher level have higher richness. If the profile for one site is everywhere above the profile for another site, then this means that the site with the highest profile is the more diverse of the two and when curves for communities intersect, this mean that they cannot be ranked [37]. For these measurements we calculated the diversity metrics for a randomly selected subset of 2,288 sequences per soil, as alpha diversity indices are correlated with the number of sequences and the same number of sequences per sample is recommended [38].

Beta diversity was analyzed by using Principal Coordinates Analysis (PCoA) which is an ordination method based on multivariate statistical analysis that maps the samples in different dimensions and reflects the similarity of the biological communities. A matrix using the UniFrac metric (weighted and unweighted) for each pair of environments was calculated. The distances were turned into points in space with the number of dimensions one less than the number of samples. The first three principal dimensions were used to plot a three-dimensional graph that was visualized using KING [39]. To test whether the results were robust to sample size we used a sequence-jackknifing technique in which the PCoA clusters were regenerated using a subset of 1716 sequences (corresponding to about 74% of the total number of sequences obtained in the sample with the smallest number of sequences) randomly selected from each soil for 100 replicate trials. The Jackknifed PCoA was performed using QIIME [30]. The clusters observed in the PCoA were confirmed by a similarity percentage analysis (SIMPER) [40]. The SIMPER analysis was performed at the family level because the Good’s coverage indicated that at this taxonomic level the samples were well represented by the number of sequences obtained (Table 1). The SIMPER performs pairwise comparisons of groups of sampling units and finds the average contributions of each OTU to the average overall Bray-Curtis dissimilarity between samples through the decomposition of Bray-Curtis dissimilarity. The decomposition of dissimilarity is calculated by the difference of abundance of each OTU in each sample. The weighted OTU table obtained as described above was transformed using log (x +1) to normalize data and the SIMPER analysis was run using R [27] through the vegan package V.2.0–5 [41] with a 70% cutoff. Due to the need for intense coverage for this type of analysis [38] the SIMPER was performed at the family level.

**Table 1:** Total number of high-quality sequences, coverage for taxonomic groups and Phylogenetic Diversity (PD).

Land use	Total number of sequences	Sequence coverage (%)						PD*
		Phylum	Class	Order	Family	Genus	3% cutoff	
AP1	8405	100	100	100	99.9	99.6	77.9	65.30
AP2	9227	100	100	99.9	99.8	99.6	80.3	60.24
AP3	7821	100	99.9	99.9	99.8	99.7	83.3	50.90
AP4	7133	100	99.9	99.9	99.8	99.6	77.3	62.16
NF1	9983	100	100	99.9	99.9	99.5	82.4	62.47
NF2	7420	100	99.9	99.9	99.8	99.5	78.4	65.19
NF3	12094	100	100	100	99.9	99.6	81.4	71.51
NF4	11318	100	100	100	99.9	99.7	82.4	67.32
NP1	6770	100	99.9	99.9	99.7	99.5	79.7	60.80
NP2	3949	100	99.9	99.8	99.5	98.7	70.9	64.96
NP3	2554	99.9	99.8	99.7	99.0	98.3	99.9	63.90
NP4	2288	99.9	99.7	99.6	99.3	98.2	99.9	63.81
SF1	10472	100	100	100	99.9	99.6	79.8	70.28
SF2	14358	100	100	99.2	99.9	99.6	81.7	71.04
SF3	10248	100	100	100	99.9	99.6	78.8	69.31
SF4	16367	100	100	100	99.9	99.8	87.1	61.79

* The means did not differ statistically between the samples by the Tukey test at 5% probability error. The PD was calculated at the family level. AP = Acacia plantation; NF = Natural forest; NP = Natural pasture; SF = Soybean field.

### Distribution of Unique and Shared OTUs and Contribution of OTUs to Dissimilarity Across Distinct Land Uses

Network-based analysis [30] was applied to examine the OTUs (family level) shared among the soil samples. The network allows for the visualization of the OTUs that are either unique or shared by specific groups of soil samples. To obtain reliable results, this approach must have intense coverage [36] or must be applied after removing the singletons. Connections were drawn between samples and OTUs, and the network was arranged in a neat looking diagram to show the distribution of OTUs over the environments. The diagram was generated with Cytoscape [42] with two kinds of nodes; OTU-nodes and soil sample nodes. The OTUs found in only one treatment were connected by only one line (named edge) and the OTUs found in more than one treatment were connected by more than one edge.

### Measurement of Microbial Metabolic Activity

Three composite samples from each land-use, collected as described above, were used to measure the microbial metabolic activity. The microbial metabolic activity was estimated by measuring the microbial biomass carbon (MBC) and by calculating the metabolic quotient (qCO_2_). The estimation of microbial biomass carbon was conducted by fumigation-extraction method [43] and the metabolic quotient was calculated by the ratio between the basal respiration and the microbial biomass [44]. Microbial biomass carbon and metabolic quotient among different land-uses were compared by Tukey’s test at p≤0.05.

## Results

### Soil Physicochemical Properties

The physicochemical properties of soils from the four land uses are presented in the Table S1. Although the land uses were in the same soil type, differences in almost all edaphic properties were observed Soils from acacia plantation, soybean field and natural pasture were more similar to each other than the soil from the natural forests (Figure S2 and Table S1). The percentage of clay in the natural forest was about 1.6-fold higher than in the other soils. Natural forest presented higher level of P, Ca, Mg, Zn nutrients, higher cation exchange capacity (CEC), higher base saturation (BS) and lower K nutrient than other sites. Potassium concentration of acacia plantation, soybean field and natural forest were similar.

### Composition and Distribution of Soil Bacterial Communities

A total of 155,195 raw sequence reads were obtained in this study. The number of high- quality sequences obtained after sequence processing in each sample and the sequence coverage is presented in Table 1. A total of 140,407 high-quality sequences longer than 200 bp were assigned to the Bacteria domain and 80.8% of these sequences were classified below the domain level. An average of 8,775 sequences (≥200 bases) were obtained per sample representing coverage of 99% up to family level. The coverage indicated that we could perform the following OTU-based analysis at the family level but not at lower taxonomical levels.

Within the classified sequences, a total of 19 phyla were found within the samples. The dominant phyla within the samples were Proteobacteria (34.4% ±2.3%), Acidobacteria (20.8% ±5.2%), Actinobacteria (11.6% ±4.0%), Bacteroidetes (3.5% ±1.6%), Verrucomicrobia (3.5% ±1.2%), Firmicutes (2.1% ±0.9%), Gemmatimonadetes (1.2% ±0.6%) and Planctomycetes (1.1% ±0.6%). The phyla with relative abundance smaller than 1% were considered as rare (Table S2). They were BRC1, Chloroflexi, Deinococcus-Thermus, Nitrospira, OD1, OP10, OP11, TM7 and WS3. The phyla BRC1 and Deinococcus-Thermus were found only in the natural pasture soil.

### Soil Bacterial Diversity and Similarity Based on Membership and Structure

**Figure 1:**
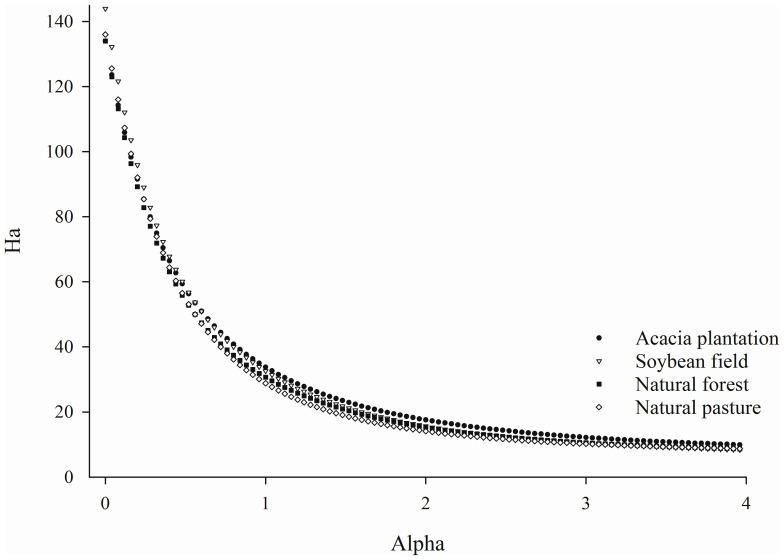
Rényi diversity profiles of soil bacterial communities from different land uses. The x-axis shows the α value of the Rényi formula and y-axis shows Rényi diversity profiles values (Hα). α values at the scales of 0, 1, 2 are related to species richness, Shannon diversity index and Simpson diversity, respectively.

**Figure 2:**
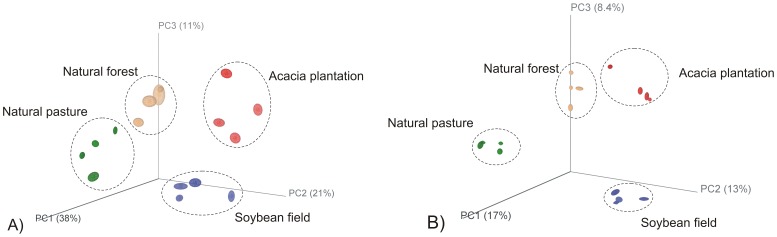
Jackknifed Principal Coordinates plot (PCoA) depicting the clusters of bacterial communities within the soil sample from four land-uses in biome Pampa. (A) Weighted UniFrac distance metrics; (B) Unweighted UniFrac distance metric. The clusters were generated using a subset of 1716 sequences from each environment. The positions of the points are the average for the jackknife replicates and ellipses around points represent the interquartile range (IQR) for the 1000 jackknife replicates.

In order to identify shifts related to bacterial diversity between land uses two different metrics to calculate bacterial diversity among samples were applied: Phylogenetic Diversity (PD) (Table 1) and Rényi diversity profiles (Figure 1). Both measures indicated that the samples presented similar bacterial diversity. The Phylogenetic Diversity did not differed statistically between samples by the Tukey test at 5% probability error and, according to the Rényi profiles, the samples presented similar degree of richness, diversity and evenness. Once no diversity differences were found among samples, we next attempted to determine whether the land-use caused shifts in the structure of the bacterial communities. To assess those differences we applied a Jackknifed PCoA analysis. Four well-defined clusters were observed for both weighted (Figure 2A) and unweighted (Figure 2B) UniFrac distance metric according to the land-use. The relatively little variation (38.4%) was explained by the first three axes with Jackknifed unweighted PCoA. On the other hand, the first three axis of the weighted Jackknifed PCoA accounted for 70% of the variation, indicating that the overall differences between the clusters were more related to the abundance of specific OTUs than to their presence or absence. In this case, the interquartile ranges (IQRs) showed that the results were robust to sample size and evenness. For both unweighted and weighted PCoA, acacia plantation and soybean field was more associated to each other than to other land-uses studied.

### Impact of Land Use on Bacterial Groups

**Figure 3:**
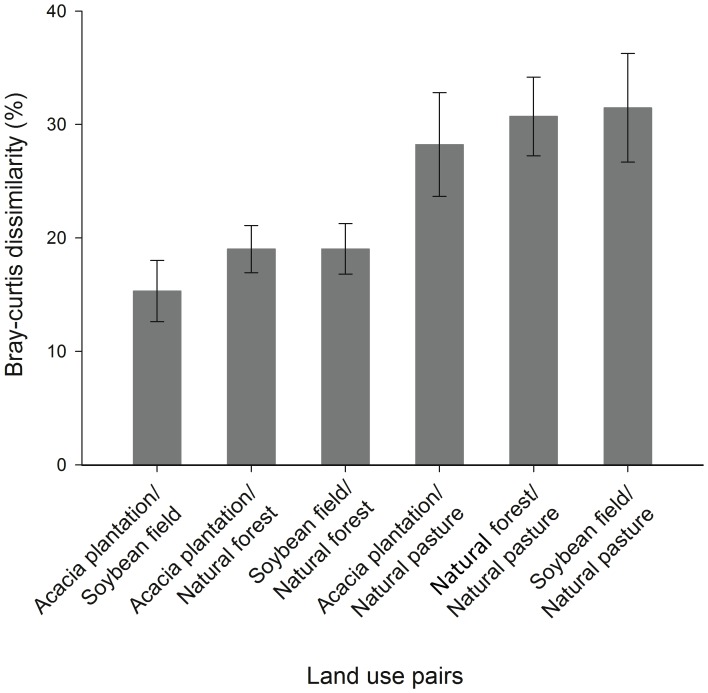
Comparison of community structure between land use pairs by similarity percentage (SIMPER) analyses. Bars represent the standard error.

Total dissimilarity between pairs of land uses and the relative contribution of each bacteria family to the observed dissimilarity was determined by SIMPER analysis. An important component of this analysis was to identify those bacteria that were responsible for the differences observed among soil samples. The total dissimilarity among all land use pairs is shown in Figure 3. On average, natural pasture presented the greatest dissimilarity (approximately 30%) among land uses. Acacia plantation and soybean field were the least dissimilar land uses presenting about 15% dissimilarity between each other. The OTUs that contributed with the community dissimilarities between the land uses are given in Table S3. Within all the bacteria family, 69% (125 OTUs out of 180) were found to consistently contribute to at least 70% to the dissimilarity in the pairwise comparisons between land uses. The SIMPER analysis also indicated that the overall differences between samples were due to a range of taxa, each contributing with a relatively small percentage of the differences. Each individual bacteria family contributed with no more than 2.5% of the total dissimilarity (Table S3).

### Shared Microbial Community

**Figure 4:**
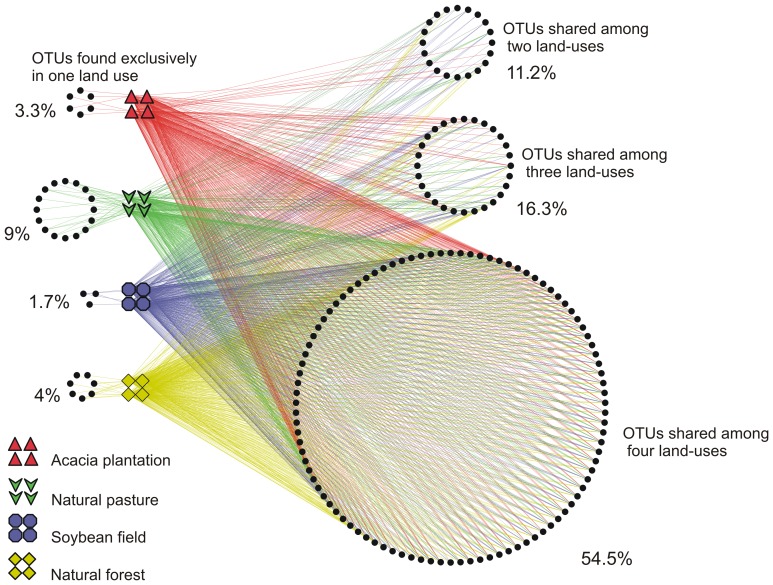
OTU network map showing OTU interactions at family level between all samples from different land-uses. The circles represent the bacterial OTUs. When one OTU is found in only one node (land-uses or soil types) the two nodes are connected with a line (an “edge”) and when one OTU is found in more than one node (land-uses or soil types) the OTU is connected with more than one edge.

Furthermore, after analyzing the differences between soil bacterial communities in each land-use, the occurrence of the bacteria families within land uses were explored using a network-like Venn diagram. Network-based analysis is used to display and analyze how OTUs are partitioned between samples. More than half of all families were present in all land-uses whereas only very few families were present exclusively in a single land-use (Figure 4). These results were in agreement with the PCoA, which indicated that the greatest alteration caused by land-use was related to the difference in the abundance of bacterial OTUs instead of presence/absence of them.

### Impact of Land Use on Microbial Biomass and Activity

Microbial biomass carbon (BMC) content and metabolic quotient differed significantly (p<0.05) among the land uses (Table 2). MBC ranged from 23.34 to 53.35 mg kg^−1^ of C and increased significantly from the human managed areas to the natural areas. Natural forest presented the highest amount of MBC (53.35 mg kg^−1^ of C) while soybean field presented the lowest amount of MBC (23.34 mg kg^−1^ of C). The highest metabolic quotient value was found in the soybean field and acacia plantation while the natural areas presented smaller metabolic quotients (Table 2).

**Table 2:** Microbial biomass C (MBC) and metabolic quotient (qCO_2_) from different land uses under the same soil type from the Brazilian Pampa biome.

Land-uses	MBC	qCO_2_
	mg kg^−1^of C	mg mg^−1^ of C-CO_2_
Acacia plantation	33.04 b	0.17 bc
Soybean field	23.34 c	0.21 a
Natural forest	53.35 a	0.11 b
Natural pasture	28.03 ab	0.08 c

Means followed by the same letter did not differ statistically between the samples by the Tukey test at 5% probability error.

## Discussion

Soil is one of the most difficult environment to work with due to its complexity, therefore there are additional methodological challenges from soil sampling to sequencing analysis [45]. Our results represent a single time point and variations in plant growth cycles and time cannot be considered. Though seasonal dynamics might affect the microbial structure and abundance, previous studies have shown that long-term patterns within these microbial communities are expected to remain generally intact [46].

Our field study is classified as observational in which randomization is restricted solely to selecting samples from the population of interest and no manipulation of experimental conditions is performed. The approach used in our experiment sometimes generates controversy among the scientific community that indicates a specific limitation regarding the statistical concept of repetitions. Some researchers argue that this type of experiment do not have true landscape level of replication but rather pseudoreplication, therefore it would not be a statistically valid experimental design for testing hypothesis. These current view gained popularity ever since the work published by Hurlbert [47], whose released his review and critique to ecologists, in matters of misconceptions in experimental design and statistical treatments emphasizing the need of genuine replication. However, after the publication of this study, the critical reevaluation of pseudoreplication has been discussed in a significant number of scientific articles. Schank and Koehnle [48] support the idea that pseudoreplication is a pseudoproblem that sets impossible standards for the majority of the experimental designs and analysis of experiments. Similarly, Oksanen [41] assume that in some of natural systems the central questions can be tackled in restricted spatial and temporal scales and the scale of ecological research should not be dictated by statistical constraints. Hargrove and Pickering [49] argue that, true landscape-level experiments are often not possible and the nature of landscape-scale studies precludes replications in the way they are constructed in classical experimentation. Our experiment, as case study, provided a unique opportunity to investigate the effects of different land uses on bacterial community among areas that are otherwise nearly identical in terms of physiography and microclimate. With points made above, the strategy thus supported our conclusions allowing us to distinguish the effect of land use on soil microbial community in the Pampa biome in local scale.

The link among plants, soil properties and belowground communities are often described as complex drivers of the ecosystem functions and any modification of this relationship might affect the microbial structure and the ecological processes [50]. With all analysis that we performed here to detect differences in soil microbial community, we found that microbial communities in the four land-uses were similar, but not identical. In our study, land-use did not affect the soil bacterial diversity, richness and evenness in natural and non-natural areas. Even using two different methods to estimate bacterial diversity, we verified no significant effect of land-use on soil bacterial diversity. Furthermore, it was not possible to detect differences in species richness and evenness. However, it is also important to recognize that similarity or difference in diversity does not mean that the species identity are the same: the same diversity might be indicative that the soil bacterial communities under the influence of environmental change will gradually being replaced by another community composed by different species that survive better within the new conditions (herein called substitution hypothesis). While it is clear that plants influence microbial community structure in soil immediately adjacent to plant roots, there is conflicting evidence about plant influences in the bulk soil across individual fields [8], [11], [13], [18], [19], [20]. In fact the soil bacterial diversity might be relatively insensitive to different land-use types [18]. Other factors might influence microbial community diversity acting independently and/or synergistically with the aboveground vegetation and soil chemical properties.

According to Nacke et al [6] and Osborne et al [51], shifts in composition and structure of bacterial communities are directly determined by land-use because of the differences related to the dominant plant community and soil chemical composition. Due to the differences in vegetation composition and soil properties across the land uses analyzed in our experiment, a clear discrimination between the microbial community structures would be expected. The small differences found in the structure of the bacterial communities might be explained by mainly four different hypothesis: i) The existence of a well adapted soil bacterial community largely independent of the specific vegetation and modifications on edaphic properties related to the land-use [52]. This view is supported by some works reporting that microbial communities are resistant to changes in plant composition 6,53,54 and can exhibit a great level of similarity despite some modifications in soil chemical properties [25]. The large amount of OTUs shared between the environments of these microbial communities might suggest the presence of a core microbial community that does not suffer any change related to land-use or edaphic properties. Identifying a soil core microbiome (the suite of members shared among microbial consortia from similar habitats) is crucial to appreciate the established microbial consortium, which is not usually subjected to change and, hence, possibly resistant/resilient to disturbances and a varying soil context [55]; ii) The bacterial community is more controlled by historical contingencies (e.g. prevalence of any type of vegetation, weather conditions) than by contemporary disturbances [56], [57]; iii) It is likely that the total bacterial community has been determined primarily by the soil type. Soil type has been indicated as a dominant factor driving microbial community composition, suggesting that certain characteristics of soils can lead to overall similarities and dissimilarities [19], [58]; iv) The apparent resilience or resistance to disturbance might be explained by cell dormancy [59]. Only a fraction of bacteria recovered from environmental samples appear to be metabolically active. Lennon and Jones [60] found that the proportion of inactive bacterial cells from soils ranged from 61 to 96%. Although it still needs to be confirmed, the significant variation of the microbial metabolic activity observed in our experiment is consistent with this hypothesis. This reflects the concept of “everything is everywhere” but most microorganisms are just everywhere albeit inactive.

An important result of our study is that microbial biomass and potential activity varied across the land uses. This difference is expected because the sampling sites differed widely in terms of soil chemical properties, plant cover and management history, factors that determine microbial metabolism. While the microbial biomass controls many important functions in soil and can be used as an indicator of environmental disturbance, the metabolic quotient (qCO2) is an index that expresses the soil quality, representing the efficiency in which organisms use the ecosystem resources [61]. Our results indicated that structural similarity were not reflected in differences in community function, supporting the idea that the coupling between functioning and composition of bacterial communities is not necessarily tight. According to Frossard et al. [62], four different outcomes are possible in such studies that contrast the structure and activity of the microbial community. One outcome is that only ecosystem function but not community structure respond to a disturbance, suggesting greater sensitivity of ecosystem function than community structure. In accordance with our conclusion, field studies that have indirectly manipulated microbial communities have not typically found evidence for strong relationships between community structure and rates of ecosystem processes [63]. Based on that, studies on microbial assemblages need to consider similarities between communities and not only focus on dissimilarities like the majority of studies performed until now.

Although the soil bacterial community did not suffer great differentiation after removing the natural vegetation and introducing agricultural crops or silviculture, we were able to detect shifts in the presence/absence of few specific bacterial groups in each land-use. These groups were not abundant but collectively represented a large part of the differences observed among samples. It is possible that only specific bacterial groups respond to changes in the aboveground vegetation, and these groups would have a low abundance in soils [64]. Since the selected sites were characterized by the same soil type and same weather conditions, we consider that the land use change and the new plant community may be the main factor responsible for alteration of these specific bacterial groups. This is according to several studies that have demonstrated that soil bacterial communities are driven by changes in land-use, including modification in plant community and soil characteristics [5], [17]. The identification of a number of specific bacterial OTUs in which distribution and abundance differ between land-uses is particularly important because it provides an experimental approach linking changes in environmental characteristics to specific bacterial groups and may help to ascertain the functional roles or environmental niches occupied by microorganisms.

## Supporting Information

Figure S1 **Brazilian Pampa biome in Rio Grande do Sul State (A) with four different land use (Natural forest, Natural pasture, Soybean field and Acacia plantation) (B).**
(TIF)

Figure S2 **PCA analysis of soil factors listed in Table S1 in four different land use (Natural forest, Natural pasture, Soybean field and Acacia plantation).**
(JPG)

Table S1 **Physical and chemical properties of subsurface soil (0–5 cm) from different land uses upon the same soil type in the Pampa biome.**
(DOC)

Table S2 **Taxon assignments for soil samples for different land-use in Pampa biome, Brazil.**
(XLS)

Table S3 **Comparison of community structure between land use pairs under the same soil type by similarity percentage (SIMPER) analyses.**
(DOC)
