# Supplementary figures and images for: Soil-Borne Bacterial Structure and Diversity Does Not Reflect Community Activity in Pampa Biome
Source: PLoS One. 2013 Oct 16;8(10):e76465. doi: 10.1371/journal.pone.0076465 (PMC3797755; doi:10.1371/journal.pone.0076465)

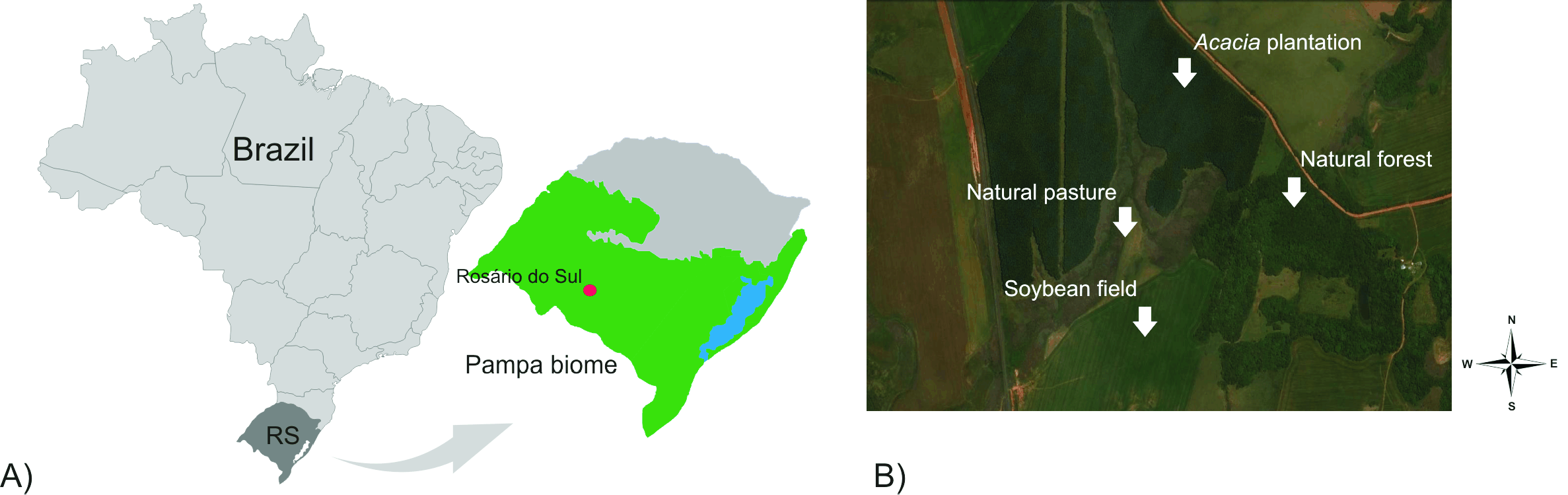

Supplement: Figure S1 — Brazilian Pampa biome in Rio Grande do Sul State (A) with four different land use (Natural forest, Natural pasture, Soybean field and Acacia plantation) (B). (TIF) [file pone.0076465.s001.tif]

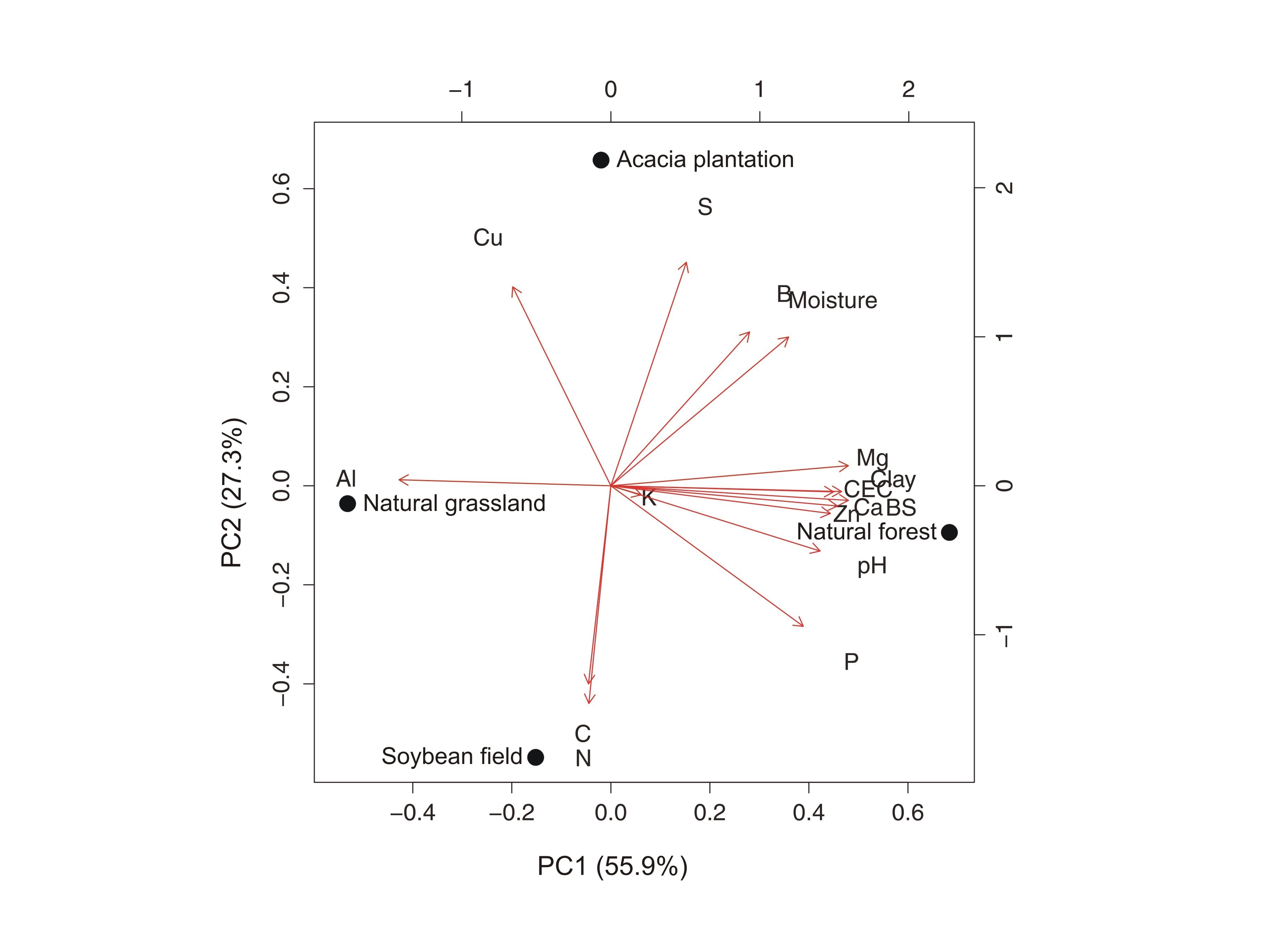

Supplement: Figure S2 — PCA analysis of soil factors listed in Table S1 in four different land use (Natural forest, Natural pasture, Soybean field and Acacia plantation). (JPG) [file pone.0076465.s002.jpg]
