# Supplementary material for: Soil-Borne Bacterial Structure and Diversity Does Not Reflect Community Activity in Pampa Biome
Source: PLoS One. 2013 Oct 16;8(10):e76465. doi: 10.1371/journal.pone.0076465 (PMC3797755; doi:10.1371/journal.pone.0076465)
Supplement: Table S1 — Physical and chemical properties of subsurface soil (0–5 cm) from different land uses upon the same soil type in the Pampa biome. (DOC) [file pone.0076465.s003.doc]

**Table S1.**

| **Soil properties** | **Acacia**  **plantation** | **Soybean**  **field** | **Natural**  **forest** | **Natural**  **pasture** |
| --- | --- | --- | --- | --- |
| **Moisture (%)** | 20.8 | 9.7 | 19.6 | 7.1 |
| **Clay (%)** | 16.0 | 15.0 | 24.0 | 14.0 |
| **pH** | 6.0 | 6.1 | 6.2 | 5.8 |
| **P (cmolc L^-1^)** | 4.5 | 21.8 | 32.3 | 4.5 |
| **K (cmolc L^-1^)** | 0.35 | 0.40 | 0.20 | 0.08 |
| **Al (cmolc L^-1^)** | 0.4 | 0.4 | 0.0 | 1.3 |
| **Ca (cmolc L^-1^)** | 2.36 | 1.97 | 11.3 | 1.16 |
| **Mg (cmolc L^-1^)** | 0.73 | 0.51 | 1.42 | 0.19 |
| **CEC pH7** | 7.9 | 6.8 | 16.4 | 6.9 |
| **BS (%)** | 43.8 | 42.6 | 78.8 | 20.8 |
| **Zn (mg L^-1^)** | 1.85 | 1.60 | 25.8 | 1.28 |
| **Cu (mg L^-1^)** | 3.25 | 1.20 | 0.65 | 1.68 |
| **S (mg L^-1^)** | 12.0 | 6.0 | 9.0 | 7.2 |
| **B (mg L^-1^)** | 0.7 | 0.5 | 0.6 | 0.4 |
| **TOC (%)** | 1.00 | 5.02 | 1.77 | 1.43 |
| **Total N (%)** | 0.01 | 0.39 | 0.13 | 0.11 |

P: Phosphorus; K: Potassium; Al: Aluminum; Ca: Calcium; Mg:

Magnesium; CEC: Cation exchange capacity; BS: Base saturation; Zn: Zinc; Cu: Copper; S: Sulphur; B: Boron; TOC: Total Organic Carbon; N: Total nitrogen
