# Supplementary material for: Soil-Borne Bacterial Structure and Diversity Does Not Reflect Community Activity in Pampa Biome
Source: PLoS One. 2013 Oct 16;8(10):e76465. doi: 10.1371/journal.pone.0076465 (PMC3797755; doi:10.1371/journal.pone.0076465)
Supplement: Table S3 — Comparison of community structure between land use pairs under the same soil type by similarity percentage (SIMPER) analyses. (DOC) [file pone.0076465.s005.doc]

**Table S3.** Comparison of community structure between land use pairs under the same soil type by similarity percentage (SIMPER) analyses.

| **Closest bacterial relative** | **Contribution to**  **dissimilarity** | **Proportion of**  **all sequences** | |
| --- | --- | --- | --- |
|  |  | **Acacia plantation** | **Soybean Field** |
|  | **%** | | |
| Proteobacteria;Gammaproteobacteria;Legionellales;Coxiellaceae | 1.7 | 0.04 | 0.16 |
| Bacteroidetes;Sphingobacteria;Sphingobacteriales;Other | 1.7 | 0.17 | 0.47 |
| Proteobacteria;Alphaproteobacteria;Sphingomonadales;Erythrobacteraceae | 1.69 | 0.02 | 0.11 |
| Proteobacteria;Gammaproteobacteria;Enterobacteriales;Enterobacteriaceae | 1.55 | 0.16 | 0.01 |
| Proteobacteria;Alphaproteobacteria;Rhodospirillales;Rhodospirillaceae | 1.48 | 0.01 | 0.07 |
| Nitrospira;Nitrospira;Nitrospirales;Nitrospiraceae | 1.4 | 0.29 | 0.79 |
| Bacteroidetes;Other;Other;Other | 1.29 | 0.20 | 0.33 |
| Actinobacteria;Actinobacteria;Solirubrobacterales;Patulibacteraceae | 1.22 | 0.06 | 0.01 |
| Proteobacteria;Betaproteobacteria;Other;Other | 1.21 | 0.85 | 1.72 |
| Proteobacteria;Gammaproteobacteria;Xanthomonadales;Sinobacteraceae | 1.2 | 0.19 | 0.07 |
| Actinobacteria;Actinobacteria;Actinomycetales;Micrococcaceae | 1.2 | 0.01 | 0.03 |
| Proteobacteria;Betaproteobacteria;Nitrosomonadales;Nitrosomonadaceae | 1.19 | 0.04 | 0.09 |
| OD1;OD1_genera_incertae_sedis;Other;Other | 1.1 | 0.02 | 0.05 |
| Acidobacteria;Acidobacteria_Gp4;Gp4;Other | 1.09 | 1.38 | 2.77 |
| Unclassified;Other;Other;Other;Other | 1.06 | 0.04 | 0.10 |
| Bacteroidetes;Flavobacteria;Flavobacteriales;Flavobacteriaceae | 1.06 | 0.05 | 0.06 |
| Actinobacteria;Actinobacteria;Actinomycetales;Geodermatophilaceae | 1.04 | 0.10 | 0.24 |
| Actinobacteria;Actinobacteria;Actinomycetales;Nakamurellaceae | 1.03 | 0.02 | 0.06 |
| Proteobacteria;Alphaproteobacteria;Rhizobiales;Methylobacteriaceae | 1.02 | 0.11 | 0.18 |
| Firmicutes;Clostridia;Clostridiales;Other | 1.01 | 0.03 | <0.01 |
| Actinobacteria;Actinobacteria;Actinomycetales;Nocardiaceae | 1.01 | 0.03 | 0.02 |
| Verrucomicrobia;Verrucomicrobiae;Verrucomicrobiales;Verrucomicrobiaceae | 0.99 | 0.03 | 0.04 |
| Proteobacteria;Gammaproteobacteria;Xanthomonadales;Xanthomonadaceae | 0.97 | 0.55 | 0.86 |
| Proteobacteria;Betaproteobacteria;Burkholderiales;Burkholderiales_incertae_sedis | 0.97 | 0.35 | 0.51 |
| Firmicutes;Clostridia;Clostridiales;Clostridiaceae | 0.95 | <0.01 | 0.02 |
| Actinobacteria;Actinobacteria;Actinomycetales;Nocardioidaceae | 0.94 | 0.57 | 0.31 |
| Proteobacteria;Gammaproteobacteria;Legionellales;Legionellaceae | 0.94 | 0.03 | 0.06 |
| Proteobacteria;Deltaproteobacteria;Myxococcales;Myxococcaceae | 0.93 | 0.02 | 0.02 |
| Acidobacteria;Acidobacteria_Gp7;Gp7;Other | 0.92 | 0.26 | 0.48 |
| Proteobacteria;Gammaproteobacteria;Pseudomonadales;Pseudomonadaceae | 0.9 | 0.06 | 0.01 |
| Verrucomicrobia;Opitutae;Opitutales;Opitutaceae | 0.89 | 0.15 | 0.19 |
| Proteobacteria;Deltaproteobacteria;Desulfuromonadales;Geobacteraceae | 0.89 | <0.01 | 0.02 |
| Proteobacteria;Betaproteobacteria;Burkholderiales;Other | 0.88 | 0.43 | 0.67 |
| Proteobacteria;Betaproteobacteria;Rhodocyclales;Rhodocyclaceae | 0.88 | <0.01 | 0.02 |
| Bacteroidetes;Sphingobacteria;Sphingobacteriales;Cytophagaceae | 0.88 | 0.06 | 0.04 |
| Bacteroidetes;Sphingobacteria;Sphingobacteriales;Sphingobacteriaceae | 0.87 | 0.25 | 0.15 |
| Actinobacteria;Actinobacteria;Acidimicrobidae_incertae_sedis;Ilumatobacter | 0.86 | 0.02 | 0.01 |
| Bacteroidetes;Sphingobacteria;Sphingobacteriales;Chitinophagaceae | 0.86 | 2.27 | 3.28 |
| Acidobacteria;Acidobacteria_Gp10;Gp10;Other | 0.85 | 0.07 | 0.07 |
| Actinobacteria;Actinobacteria;Actinomycetales;Bogoriellaceae | 0.84 | 0.02 | 0.02 |
| Firmicutes;Bacilli;Bacillales;Planococcaceae | 0.84 | 0.04 | 0.02 |
| Actinobacteria;Actinobacteria;Rubrobacterales;Rubrobacteraceae | 0.82 | <0.01 | 0.03 |
| Gemmatimonadetes;Gemmatimonadetes;Gemmatimonadales;Gemmatimonadaceae | 0.82 | 1.32 | 2.04 |
| Proteobacteria;Gammaproteobacteria;Pseudomonadales;Moraxellaceae | 0.81 | 0.16 | 0.22 |
| Actinobacteria;Actinobacteria;Actinomycetales;Cryptosporangiaceae | 0.81 | 0.03 | 0.02 |
| Actinobacteria;Actinobacteria;Actinomycetales;Propionibacteriaceae | 0.8 | 0.12 | 0.16 |
| Actinobacteria;Actinobacteria;Actinomycetales;Thermomonosporaceae | 0.78 | 0.02 | 0.02 |
| Actinobacteria;Actinobacteria;Actinomycetales;Microbacteriaceae | 0.78 | 0.34 | 0.18 |
| Acidobacteria;Acidobacteria_Gp6;Gp6;Other | 0.76 | 1.57 | 2.34 |
| Firmicutes;Bacilli;Bacillales;Other | 0.75 | 0.35 | 0.39 |
| Firmicutes;Erysipelotrichi;Erysipelotrichales;Erysipelotrichaceae | 0.74 | <0.01 | 0.01 |
| Acidobacteria;Acidobacteria_Gp16;Gp16;Other | 0.74 | 0.33 | 0.38 |
| Proteobacteria;Alphaproteobacteria;Sphingomonadales;Sphingomonadaceae | 0.74 | 4.12 | 5.58 |
| Proteobacteria;Deltaproteobacteria;Bdellovibrionales;Bdellovibrionaceae | 0.73 | 0.02 | 0.03 |
| Actinobacteria;Actinobacteria;Solirubrobacterales;Solirubrobacteraceae | 0.73 | 0.40 | 0.17 |
| Proteobacteria;Alphaproteobacteria;Rhodospirillales;Other | 0.71 | 0.16 | 0.18 |
| Proteobacteria;Deltaproteobacteria;Other;Other | 0.68 | 0.57 | 0.85 |
| Proteobacteria;Deltaproteobacteria;Myxococcales;Phaselicystidaceae | 0.68 | <0.01 | 0.01 |
| Actinobacteria;Actinobacteria;Actinomycetales;Kineosporiaceae | 0.68 | 0.04 | 0.04 |
| Actinobacteria;Actinobacteria;Actinomycetales;Intrasporangiaceae | 0.68 | 0.57 | 0.63 |
| Actinobacteria;Actinobacteria;Solirubrobacterales;Conexibacteraceae | 0.68 | 0.14 | 0.05 |
| Acidobacteria;Acidobacteria_Gp3;Gp3;Other | 0.65 | 6.12 | 7.79 |
| Proteobacteria;Deltaproteobacteria;Myxococcales;Cystobacteraceae | 0.65 | 0.29 | 0.37 |
| Firmicutes;Other;Other;Other | 0.65 | 0.02 | <0.01 |
| Chloroflexi;Anaerolineae;Anaerolineales;Anaerolineaceae | 0.65 | <0.01 | 0.01 |
| Verrucomicrobia;Subdivision3;Subdivision3_genera_incertae_sedis;Other | 0.65 | 0.71 | 0.90 |
| Actinobacteria;Actinobacteria;Actinomycetales;Streptosporangiaceae | 0.65 | 0.01 | 0.01 |
| Verrucomicrobia;Other;Other;Other | 0.64 | 0.31 | 0.32 |
| Proteobacteria;Alphaproteobacteria;Rhizobiales;Methylocystaceae | 0.64 | 0.04 | 0.02 |
| Bacteria_incertae_sedis;Ktedonobacteria;Ktedonobacterales;Ktedonobacteraceae | 0.64 | 0.87 | 0.59 |
| Actinobacteria;Actinobacteria;Actinomycetales;Actinospicaceae | 0.63 | <0.01 | 0.01 |
| Proteobacteria;Alphaproteobacteria;Rhizobiales;Rhizobiaceae | 0.63 | 0.07 | 0.06 |
| Verrucomicrobia;Spartobacteria;Spartobacteria_genera_incertae_sedis;Other | 0.63 | 1.88 | 2.25 |
| **Closest bacterial relative** | **Contribution to**  **dissimilarity** | **Proportion of**  **all sequences** | |
|  |  | **Acacia plantation** | **Natural forest** |
|  | **%** | | |
| Acidobacteria;Acidobacteria_Gp25;Gp25;Other | 2.23 | <0.01 | 0.16 |
| Acidobacteria;Acidobacteria_Gp17;Gp17;Other | 2.15 | <0.01 | 0.17 |
| Acidobacteria;Acidobacteria_Gp11;Gp11;Other | 2.09 | <0.01 | 0.12 |
| Bacteroidetes;Flavobacteria;Flavobacteriales;Flavobacteriaceae | 1.91 | 0.05 | 0.39 |
| Acidobacteria;Acidobacteria_Gp15;Gp15;Other | 1.85 | <0.01 | 0.09 |
| Acidobacteria;Acidobacteria_Gp22;Gp22;Other | 1.78 | <0.01 | 0.08 |
| Actinobacteria;Actinobacteria;Actinomycetales;Geodermatophilaceae | 1.76 | 0.10 | <0.01 |
| Nitrospira;Nitrospira;Nitrospirales;Nitrospiraceae | 1.71 | 0.29 | 1.81 |
| Actinobacteria;Actinobacteria;Actinomycetales;Propionibacteriaceae | 1.61 | 0.12 | 0.65 |
| Bacteroidetes;Other;Other;Other | 1.53 | 0.20 | 0.70 |
| Proteobacteria;Alphaproteobacteria;Rhizobiales;Methylobacteriaceae | 1.52 | 0.11 | <0.01 |
| Bacteria_incertae_sedis;Ktedonobacteria;Ktedonobacterales;Ktedonobacteraceae | 1.51 | 0.87 | 0.10 |
| Acidobacteria;Acidobacteria_Gp5;Gp5;Other | 1.39 | 0.24 | 1.12 |
| Firmicutes;Bacilli;Bacillales;Other | 1.34 | 0.35 | 0.06 |
| Acidobacteria;Acidobacteria_Gp6;Gp6;Other | 1.32 | 1.57 | 6.39 |
| Acidobacteria;Acidobacteria_Gp7;Gp7;Other | 1.19 | 0.26 | 1.05 |
| Actinobacteria;Actinobacteria;Actinomycetales;Catenulisporaceae | 1.18 | 0.07 | 0.01 |
| Actinobacteria;Actinobacteria;Actinomycetales;Microbacteriaceae | 1.18 | 0.34 | 0.05 |
| Bacteroidetes;Sphingobacteria;Sphingobacteriales;Sphingobacteriaceae | 1.14 | 0.25 | 0.05 |
| Proteobacteria;Deltaproteobacteria;Other;Other | 1.11 | 0.57 | 2.20 |
| Proteobacteria;Alphaproteobacteria;Rhodospirillales;Acetobacteraceae | 1.11 | 1.49 | 0.29 |
| Proteobacteria;Betaproteobacteria;Burkholderiales;Burkholderiaceae | 1.09 | 0.52 | 0.09 |
| Bacteroidetes;Sphingobacteria;Sphingobacteriales;Other | 1.07 | 0.17 | 0.37 |
| OP10;OP10_genera_incertae_sedis;Other;Other | 1 | 0.10 | 0.01 |
| Actinobacteria;Actinobacteria;Actinomycetales;Intrasporangiaceae | 1 | 0.57 | 0.12 |
| Proteobacteria;Betaproteobacteria;Burkholderiales;Other | 0.98 | 0.43 | 1.21 |
| Acidobacteria;Acidobacteria_Gp4;Gp4;Other | 0.98 | 1.38 | 3.65 |
| Chloroflexi;Anaerolineae;Anaerolineales;Anaerolineaceae | 0.98 | <0.01 | 0.03 |
| Verrucomicrobia;Verrucomicrobiae;Verrucomicrobiales;Verrucomicrobiaceae | 0.96 | 0.03 | 0.07 |
| Planctomycetes;Planctomycetacia;Planctomycetales;Planctomycetaceae | 0.95 | 1.44 | 0.53 |
| Proteobacteria;Gammaproteobacteria;Legionellales;Coxiellaceae | 0.94 | 0.04 | 0.10 |
| Proteobacteria;Gammaproteobacteria;Pseudomonadales;Pseudomonadaceae | 0.94 | 0.06 | 0.13 |
| Actinobacteria;Actinobacteria;Solirubrobacterales;Patulibacteraceae | 0.92 | 0.06 | 0.02 |
| Acidobacteria;Acidobacteria_Gp16;Gp16;Other | 0.9 | 0.33 | 0.70 |
| Proteobacteria;Gammaproteobacteria;Xanthomonadales;Sinobacteraceae | 0.88 | 0.19 | 0.33 |
| Proteobacteria;Betaproteobacteria;Other;Other | 0.85 | 0.85 | 1.63 |
| Actinobacteria;Actinobacteria;Actinomycetales;Kineosporiaceae | 0.85 | 0.04 | 0.01 |
| Proteobacteria;Betaproteobacteria;Nitrosomonadales;Nitrosomonadaceae | 0.85 | 0.04 | <0.01 |
| Firmicutes;Bacilli;Bacillales;Bacillaceae | 0.84 | 2.30 | 0.64 |
| Proteobacteria;Deltaproteobacteria;Desulfuromonadales;Geobacteraceae | 0.82 | <0.01 | 0.03 |
| WS3;WS3_genera_incertae_sedis;Other;Other | 0.82 | <0.01 | <0.01 |
| Proteobacteria;Gammaproteobacteria;Pseudomonadales;Moraxellaceae | 0.81 | 0.16 | 0.12 |
| Proteobacteria;Gammaproteobacteria;Enterobacteriales;Enterobacteriaceae | 0.8 | 0.16 | 0.08 |
| Unclassified;Other;Other;Other;Other | 0.79 | 0.04 | 0.04 |
| Actinobacteria;Actinobacteria;Actinomycetales;Nocardiaceae | 0.79 | 0.03 | 0.03 |
| Proteobacteria;Alphaproteobacteria;Sphingomonadales;Other | 0.79 | 0.09 | 0.01 |
| Acidobacteria;Acidobacteria_Gp10;Gp10;Other | 0.79 | 0.07 | 0.10 |
| Bacteroidetes;Sphingobacteria;Sphingobacteriales;Cytophagaceae | 0.79 | 0.06 | 0.09 |
| Actinobacteria;Actinobacteria;Solirubrobacterales;Conexibacteraceae | 0.78 | 0.14 | 0.06 |
| Proteobacteria;Betaproteobacteria;Burkholderiales;Burkholderiales_incertae_sedis | 0.75 | 0.35 | 0.11 |
| Chloroflexi;Thermomicrobia;Other;Other | 0.74 | <0.01 | <0.01 |
| Proteobacteria;Gammaproteobacteria;Xanthomonadales;Xanthomonadaceae | 0.73 | 0.55 | 0.91 |
| Actinobacteria;Actinobacteria;Actinomycetales;Mycobacteriaceae | 0.72 | 1.05 | 0.35 |
| Firmicutes;Bacilli;Bacillales;Paenibacillaceae | 0.71 | 0.19 | 0.07 |
| Actinobacteria;Actinobacteria;Actinomycetales;Nakamurellaceae | 0.7 | 0.02 | 0.03 |
| Actinobacteria;Actinobacteria;Actinomycetales;Pseudonocardiaceae | 0.69 | 0.19 | 0.03 |
| Actinobacteria;Actinobacteria;Actinomycetales;Cryptosporangiaceae | 0.68 | 0.03 | <0.01 |
| Actinobacteria;Actinobacteria;Actinomycetales;Nocardioidaceae | 0.68 | 0.57 | 0.23 |
| Proteobacteria;Betaproteobacteria;Burkholderiales;Oxalobacteraceae | 0.67 | 0.19 | 0.06 |
| Acidobacteria;Acidobacteria_Gp13;Gp13;Other | 0.67 | 0.03 | 0.04 |
| Actinobacteria;Actinobacteria;Actinomycetales;Thermomonosporaceae | 0.66 | 0.02 | 0.02 |
| Verrucomicrobia;Opitutae;Opitutales;Opitutaceae | 0.65 | 0.15 | 0.08 |
| Proteobacteria;Alphaproteobacteria;Rhizobiales;Methylocystaceae | 0.65 | 0.04 | 0.01 |
| Proteobacteria;Gammaproteobacteria;Xanthomonadales;Other | 0.63 | 0.22 | <0.01 |
| Proteobacteria;Alphaproteobacteria;Caulobacterales;Caulobacteraceae | 0.63 | 0.53 | 0.20 |
| Actinobacteria;Actinobacteria;Acidimicrobidae_incertae_sedis;Ilumatobacter | 0.63 | 0.02 | 0.06 |
| **Closest bacterial relative** | **Contribution to**  **dissimilarity** | **Proportion of**  **all sequences** | |
|  |  | **Soybean**  **field** | **Natural forest** |
|  | **%** | | |
| Actinobacteria;Actinobacteria;Actinomycetales;Geodermatophilaceae | 2.42 | 0.24 | <0.01 |
| Proteobacteria;Alphaproteobacteria;Rhizobiales;Methylobacteriaceae | 2.12 | 0.18 | <0.01 |
| Acidobacteria;Acidobacteria_Gp25;Gp25;Other | 2.06 | <0.01 | 0.16 |
| Acidobacteria;Acidobacteria_Gp11;Gp11;Other | 1.93 | <0.01 | 0.12 |
| Proteobacteria;Betaproteobacteria;Nitrosomonadales;Nitrosomonadaceae | 1.7 | 0.09 | <0.01 |
| Acidobacteria;Acidobacteria_Gp22;Gp22;Other | 1.64 | <0.01 | 0.08 |
| Acidobacteria;Acidobacteria_Gp17;Gp17;Other | 1.64 | 0.01 | 0.17 |
| Proteobacteria;Alphaproteobacteria;Sphingomonadales;Erythrobacteraceae | 1.62 | 0.11 | 0.01 |
| Bacteria_incertae_sedis;Ktedonobacteria;Ktedonobacterales;Ktedonobacteraceae | 1.54 | 0.59 | 0.10 |
| Firmicutes;Bacilli;Bacillales;Other | 1.48 | 0.39 | 0.06 |
| Bacteroidetes;Flavobacteria;Flavobacteriales;Flavobacteriaceae | 1.46 | 0.06 | 0.39 |
| Proteobacteria;Alphaproteobacteria;Rhodospirillales;Acetobacteraceae | 1.45 | 1.68 | 0.29 |
| OP10;OP10_genera_incertae_sedis;Other;Other | 1.33 | 0.11 | 0.01 |
| Proteobacteria;Gammaproteobacteria;Xanthomonadales;Sinobacteraceae | 1.32 | 0.07 | 0.33 |
| Proteobacteria;Gammaproteobacteria;Pseudomonadales;Pseudomonadaceae | 1.31 | 0.01 | 0.13 |
| Actinobacteria;Actinobacteria;Actinomycetales;Intrasporangiaceae | 1.26 | 0.63 | 0.12 |
| Acidobacteria;Acidobacteria_Gp15;Gp15;Other | 1.24 | 0.01 | 0.09 |
| Actinobacteria;Actinobacteria;Actinomycetales;Catenulisporaceae | 1.24 | 0.06 | 0.01 |
| Proteobacteria;Betaproteobacteria;Burkholderiales;Burkholderiales_incertae_sedis | 1.21 | 0.51 | 0.11 |
| Actinobacteria;Actinobacteria;Actinomycetales;Micrococcaceae | 1.2 | 0.03 | <0.01 |
| Acidobacteria;Acidobacteria_Gp5;Gp5;Other | 1.09 | 0.21 | 1.12 |
| Proteobacteria;Alphaproteobacteria;Rhizobiales;Methylocystaceae | 1.09 | 0.02 | 0.01 |
| Proteobacteria;Alphaproteobacteria;Sphingomonadales;Sphingomonadaceae | 1.08 | 5.58 | 1.66 |
| Proteobacteria;Alphaproteobacteria;Rhodospirillales;Rhodospirillaceae | 1.03 | 0.07 | 0.02 |
| Firmicutes;Bacilli;Bacillales;Bacillaceae | 1.02 | 2.02 | 0.64 |
| Proteobacteria;Alphaproteobacteria;Sphingomonadales;Other | 1.02 | 0.07 | 0.01 |
| Planctomycetes;Planctomycetacia;Planctomycetales;Planctomycetaceae | 1 | 1.20 | 0.53 |
| Bacteroidetes;Sphingobacteria;Sphingobacteriales;Sphingobacteriaceae | 0.98 | 0.15 | 0.05 |
| Proteobacteria;Gammaproteobacteria;Legionellales;Legionellaceae | 0.97 | 0.06 | 0.01 |
| Actinobacteria;Actinobacteria;Actinomycetales;Propionibacteriaceae | 0.96 | 0.16 | 0.65 |
| Proteobacteria;Gammaproteobacteria;Enterobacteriales;Enterobacteriaceae | 0.95 | 0.01 | 0.08 |
| Actinobacteria;Actinobacteria;Actinomycetales;Microbacteriaceae | 0.94 | 0.18 | 0.05 |
| Proteobacteria;Gammaproteobacteria;Pseudomonadales;Moraxellaceae | 0.92 | 0.22 | 0.12 |
| Proteobacteria;Betaproteobacteria;Burkholderiales;Burkholderiaceae | 0.9 | 0.31 | 0.09 |
| Acidobacteria;Acidobacteria_Gp1;Gp1;Other | 0.85 | 3.95 | 1.65 |
| TM7;TM7_genera_incertae_sedis;Other;Other | 0.81 | 0.94 | 0.40 |
| Actinobacteria;Actinobacteria;Actinomycetales;Bogoriellaceae | 0.81 | 0.02 | <0.01 |
| Verrucomicrobia;Opitutae;Opitutales;Opitutaceae | 0.81 | 0.19 | 0.08 |
| Proteobacteria;Alphaproteobacteria;Caulobacterales;Caulobacteraceae | 0.8 | 0.47 | 0.20 |
| Firmicutes;Bacilli;Bacillales;Paenibacillaceae | 0.8 | 0.15 | 0.07 |
| Firmicutes;Bacilli;Bacillales;Planococcaceae | 0.79 | 0.02 | 0.04 |
| Proteobacteria;Deltaproteobacteria;Desulfuromonadales;Geobacteraceae | 0.78 | 0.02 | 0.03 |
| Actinobacteria;Actinobacteria;Actinomycetales;Mycobacteriaceae | 0.78 | 0.81 | 0.35 |
| Bacteroidetes;Sphingobacteria;Sphingobacteriales;Cytophagaceae | 0.77 | 0.04 | 0.09 |
| WS3;WS3_genera_incertae_sedis;Other;Other | 0.75 | 0.81 | 0.35 |
| Proteobacteria;Deltaproteobacteria;Myxococcales;Cystobacteraceae | 0.75 | 0.37 | 0.17 |
| Firmicutes;Clostridia;Clostridiales;Clostridiaceae | 0.75 | 0.02 | <0.01 |
| Actinobacteria;Actinobacteria;Actinomycetales;Pseudonocardiaceae | 0.73 | 0.14 | 0.03 |
| Proteobacteria;Betaproteobacteria;Burkholderiales;Oxalobacteraceae | 0.72 | 0.17 | 0.06 |
| Actinobacteria;Actinobacteria;Actinomycetales;Nakamurellaceae | 0.71 | 0.06 | 0.03 |
| Unclassified;Other;Other;Other;Other | 0.69 | 0.10 | 0.04 |
| Actinobacteria;Actinobacteria;Acidimicrobidae_incertae_sedis;Ilumatobacter | 0.67 | 0.01 | 0.06 |
| Acidobacteria;Acidobacteria_Gp2;Gp2;Other | 0.67 | 0.56 | 1.63 |
| Actinobacteria;Actinobacteria;Actinomycetales;Nocardiaceae | 0.66 | 0.02 | 0.03 |
| Actinobacteria;Actinobacteria;Actinomycetales;Cryptosporangiaceae | 0.66 | 0.02 | <0.01 |
| Acidobacteria;Acidobacteria_Gp13;Gp13;Other | 0.66 | 0.02 | 0.04 |
| Actinobacteria;Actinobacteria;Rubrobacterales;Rubrobacteraceae | 0.65 | 0.03 | <0.01 |
| Proteobacteria;Deltaproteobacteria;Bdellovibrionales;Bdellovibrionaceae | 0.64 | 0.03 | 0.01 |
| Actinobacteria;Actinobacteria;Actinomycetales;Kineosporiaceae | 0.64 | 0.04 | 0.01 |
| Proteobacteria;Betaproteobacteria;Rhodocyclales;Rhodocyclaceae | 0.63 | 0.02 | <0.01 |
| Verrucomicrobia;Verrucomicrobiae;Verrucomicrobiales;Verrucomicrobiaceae | 0.63 | 0.04 | 0.07 |
| Acidobacteria;Acidobacteria_Gp6;Gp6;Other | 0.62 | 2.34 | 6.39 |
| Proteobacteria;Deltaproteobacteria;Myxococcales;Myxococcaceae | 0.62 | 0.02 | <0.01 |
| Gemmatimonadetes;Gemmatimonadetes;Gemmatimonadales;Gemmatimonadaceae | 0.61 | 2.04 | 0.98 |
| Actinobacteria;Actinobacteria;Solirubrobacterales;Patulibacteraceae | 0.6 | 0.01 | 0.02 |
| Firmicutes;Erysipelotrichi;Erysipelotrichales;Erysipelotrichaceae | 0.59 | 0.01 | <0.01 |
| OD1;OD1_genera_incertae_sedis;Other;Other | 0.57 | 0.05 | 0.02 |
| **Closest bacterial relative** | **Contribution to**  **dissimilarity** | **Proportion of**  **all sequences** | |
|  |  | **Acacia plantation** | **Natural grassland** |
|  | **%** | | |
| Proteobacteria;Deltaproteobacteria;Desulfuromonadales;Geobacteraceae | 2.05 | <0.01 | 0.75 |
| Actinobacteria;Actinobacteria;Solirubrobacterales;Solirubrobacteraceae | 1.95 | 0.40 | 0.02 |
| Chloroflexi;Anaerolineae;Anaerolineales;Anaerolineaceae | 1.93 | <0.01 | 0.78 |
| Actinobacteria;Actinobacteria;Actinomycetales;Intrasporangiaceae | 1.73 | 0.57 | 0.06 |
| Actinobacteria;Actinobacteria;Actinomycetales;Pseudonocardiaceae | 1.62 | 0.19 | 0.01 |
| Acidobacteria;Acidobacteria_Gp4;Gp4;Other | 1.58 | 1.38 | 0.44 |
| Proteobacteria;Gammaproteobacteria;Enterobacteriales;Enterobacteriaceae | 1.5 | 0.16 | <0.01 |
| Actinobacteria;Actinobacteria;Solirubrobacterales;Conexibacteraceae | 1.45 | 0.14 | 0.01 |
| Actinobacteria;Actinobacteria;Actinomycetales;Nocardioidaceae | 1.45 | 0.57 | 0.08 |
| Proteobacteria;Gammaproteobacteria;Xanthomonadales;Sinobacteraceae | 1.45 | 0.19 | 0.01 |
| Actinobacteria;Actinobacteria;Acidimicrobidae_incertae_sedis;Ilumatobacter | 1.42 | 0.02 | <0.01 |
| Actinobacteria;Actinobacteria;Acidimicrobiales;Iamiaceae | 1.39 | 0.65 | 0.17 |
| Proteobacteria;Alphaproteobacteria;Rhizobiales;Phyllobacteriaceae | 1.37 | 0.20 | 0.03 |
| Actinobacteria;Actinobacteria;Actinomycetales;Microbacteriaceae | 1.31 | 0.34 | 0.07 |
| Actinobacteria;Actinobacteria;Actinomycetales;Propionibacteriaceae | 1.3 | 0.12 | 0.01 |
| Proteobacteria;Alphaproteobacteria;Sphingomonadales;Sphingomonadaceae | 1.3 | 4.12 | 1.42 |
| Verrucomicrobia;Spartobacteria;Spartobacteria_genera_incertae_sedis;Other | 1.27 | 1.88 | 0.74 |
| Actinobacteria;Actinobacteria;Actinomycetales;Streptomycetaceae | 1.09 | 0.46 | 0.20 |
| Proteobacteria;Deltaproteobacteria;Syntrophobacterales;Syntrophobacteraceae | 1.08 | <0.01 | 0.15 |
| Actinobacteria;Actinobacteria;Actinomycetales;Micromonosporaceae | 1.07 | 0.52 | 0.19 |
| Actinobacteria;Actinobacteria;Solirubrobacterales;Other | 1.07 | 4.87 | 2.14 |
| Bacteroidetes;Sphingobacteria;Sphingobacteriales;Other | 1.06 | 0.17 | 0.02 |
| Proteobacteria;Alphaproteobacteria;Rhizobiales;Rhizobiaceae | 1.02 | 0.07 | 0.03 |
| Bacteroidetes;Sphingobacteria;Sphingobacteriales;Sphingobacteriaceae | 1.01 | 0.25 | 0.08 |
| Nitrospira;Nitrospira;Nitrospirales;Nitrospiraceae | 1 | 0.29 | 0.16 |
| Gemmatimonadetes;Gemmatimonadetes;Gemmatimonadales;Gemmatimonadaceae | 1 | 1.32 | 0.65 |
| Bacteroidetes;Sphingobacteria;Sphingobacteriales;Chitinophagaceae | 0.99 | 2.27 | 1.09 |
| Firmicutes;Bacilli;Bacillales;Paenibacillaceae | 0.98 | 0.19 | 0.07 |
| Proteobacteria;Alphaproteobacteria;Rhodospirillales;Other | 0.92 | 0.16 | 0.09 |
| Proteobacteria;Alphaproteobacteria;Rhizobiales;Bradyrhizobiaceae | 0.92 | 7.20 | 4.08 |
| Firmicutes;Bacilli;Bacillales;Bacillaceae | 0.92 | 2.30 | 1.21 |
| Proteobacteria;Deltaproteobacteria;Desulfuromonadales;Other | 0.9 | <0.01 | 0.11 |
| Actinobacteria;Actinobacteria;Actinomycetales;Other | 0.89 | 4.38 | 2.59 |
| Proteobacteria;Gammaproteobacteria;Pseudomonadales;Pseudomonadaceae | 0.89 | 0.06 | 0.01 |
| Actinobacteria;Actinobacteria;Other;Other | 0.89 | 1.99 | 1.02 |
| Proteobacteria;Betaproteobacteria;Burkholderiales;Comamonadaceae | 0.87 | 0.33 | 0.22 |
| Proteobacteria;Gammaproteobacteria;Pseudomonadales;Moraxellaceae | 0.86 | 0.16 | 0.10 |
| Actinobacteria;Actinobacteria;Solirubrobacterales;Patulibacteraceae | 0.85 | 0.06 | 0.01 |
| Bacteroidetes;Flavobacteria;Flavobacteriales;Flavobacteriaceae | 0.84 | 0.05 | <0.01 |
| Proteobacteria;Alphaproteobacteria;Sphingomonadales;Other | 0.83 | 0.09 | 0.02 |
| Proteobacteria;Alphaproteobacteria;Caulobacterales;Caulobacteraceae | 0.83 | 0.53 | 0.29 |
| Firmicutes;Bacilli;Bacillales;Other | 0.77 | 0.35 | 0.23 |
| Proteobacteria;Alphaproteobacteria;Rhizobiales;Methylobacteriaceae | 0.77 | 0.11 | 0.04 |
| Firmicutes;Clostridia;Clostridiales;Veillonellaceae | 0.76 | <0.01 | 0.07 |
| Bacteroidetes;Sphingobacteria;Sphingobacteriales;Cytophagaceae | 0.75 | 0.06 | 0.02 |
| Actinobacteria;Actinobacteria;Actinomycetales;Kineosporiaceae | 0.75 | 0.04 | 0.01 |
| Proteobacteria;Deltaproteobacteria;Myxococcales;Polyangiaceae | 0.75 | 1.27 | 0.70 |
| Actinobacteria;Actinobacteria;Actinomycetales;Geodermatophilaceae | 0.74 | 0.10 | 0.06 |
| Proteobacteria;Betaproteobacteria;Burkholderiales;Burkholderiaceae | 0.74 | 0.52 | 0.55 |
| Firmicutes;Clostridia;Clostridiales;Clostridiaceae | 0.71 | <0.01 | 0.06 |
| Proteobacteria;Betaproteobacteria;Burkholderiales;Burkholderiales_incertae_sedis | 0.71 | 0.35 | 0.22 |
| Bacteroidetes;Other;Other;Other | 0.7 | 0.20 | 0.47 |
| Proteobacteria;Alphaproteobacteria;Rhizobiales;Beijerinckiaceae | 0.7 | 0.18 | 0.13 |
| Planctomycetes;Planctomycetacia;Planctomycetales;Planctomycetaceae | 0.69 | 1.44 | 1.24 |
| Verrucomicrobia;Other;Other;Other | 0.69 | 0.31 | 0.22 |
| Proteobacteria;Betaproteobacteria;Nitrosomonadales;Nitrosomonadaceae | 0.68 | 0.04 | 0.01 |
| Proteobacteria;Alphaproteobacteria;Rhizobiales;Methylocystaceae | 0.68 | 0.04 | 0.25 |
| Proteobacteria;Deltaproteobacteria;Syntrophobacterales;Syntrophaceae | 0.67 | <0.01 | 0.05 |
| Proteobacteria;Alphaproteobacteria;Rhizobiales;Other | 0.65 | 7.21 | 6.79 |
| Firmicutes;Bacilli;Bacillales;Planococcaceae | 0.65 | 0.04 | 0.01 |
| Proteobacteria;Gammaproteobacteria;Methylococcales;Methylococcaceae | 0.65 | <0.01 | 0.08 |
| Proteobacteria;Gammaproteobacteria;Legionellales;Coxiellaceae | 0.65 | 0.04 | 0.21 |
| Acidobacteria;Acidobacteria_Gp6;Gp6;Other | 0.64 | 1.57 | 1.36 |
| Actinobacteria;Actinobacteria;Acidimicrobiales;Other | 0.64 | 0.28 | 0.49 |
| Acidobacteria;Acidobacteria_Gp10;Gp10;Other | 0.63 | 0.07 | 0.03 |
| Proteobacteria;Deltaproteobacteria;Desulfovibrionales;Desulfovibrionaceae | 0.63 | <0.01 | 0.06 |
| Proteobacteria;Betaproteobacteria;Burkholderiales;Other | 0.62 | 0.43 | 0.90 |
| Verrucomicrobia;Opitutae;Opitutales;Opitutaceae | 0.61 | 0.15 | 0.11 |
| Actinobacteria;Actinobacteria;Actinomycetales;Catenulisporaceae | 0.61 | 0.07 | 0.05 |
| Proteobacteria;Deltaproteobacteria;Myxococcales;Other | 0.61 | 1.44 | 1.42 |
| **Closest bacterial relative** | **Contribution to**  **dissimilarity** | **Proportion of**  **all sequences** | |
|  |  | **Soybean**  **field** | **Natural grassland** |
|  | **%** | | |
| Acidobacteria;Acidobacteria_Gp4;Gp4;Other | 1.9 | 2.77 | 0.44 |
| Bacteroidetes;Sphingobacteria;Sphingobacteriales;Other | 1.72 | 0.47 | 0.02 |
| Actinobacteria;Actinobacteria;Actinomycetales;Intrasporangiaceae | 1.65 | 0.63 | 0.06 |
| Nitrospira;Nitrospira;Nitrospirales;Nitrospiraceae | 1.56 | 0.79 | 0.16 |
| Proteobacteria;Alphaproteobacteria;Sphingomonadales;Sphingomonadaceae | 1.47 | 5.58 | 1.42 |
| Actinobacteria;Actinobacteria;Solirubrobacterales;Solirubrobacteraceae | 1.45 | 0.17 | 0.02 |
| Actinobacteria;Actinobacteria;Actinomycetales;Propionibacteriaceae | 1.44 | 0.16 | 0.01 |
| Actinobacteria;Actinobacteria;Actinomycetales;Pseudonocardiaceae | 1.41 | 0.14 | 0.01 |
| Proteobacteria;Alphaproteobacteria;Sphingomonadales;Erythrobacteraceae | 1.37 | 0.11 | <0.01 |
| Verrucomicrobia;Spartobacteria;Spartobacteria_genera_incertae_sedis;Other | 1.37 | 2.25 | 0.74 |
| Bacteroidetes;Sphingobacteria;Sphingobacteriales;Chitinophagaceae | 1.27 | 3.28 | 1.09 |
| Gemmatimonadetes;Gemmatimonadetes;Gemmatimonadales;Gemmatimonadaceae | 1.27 | 2.04 | 0.65 |
| Chloroflexi;Anaerolineae;Anaerolineales;Anaerolineaceae | 1.22 | 0.01 | 0.78 |
| Proteobacteria;Betaproteobacteria;Nitrosomonadales;Nitrosomonadaceae | 1.2 | 0.09 | 0.01 |
| Proteobacteria;Deltaproteobacteria;Desulfuromonadales;Geobacteraceae | 1.19 | 0.02 | 0.75 |
| Actinobacteria;Actinobacteria;Actinomycetales;Geodermatophilaceae | 1.16 | 0.24 | 0.06 |
| Proteobacteria;Alphaproteobacteria;Rhodospirillales;Rhodospirillaceae | 1.16 | 0.07 | 0.02 |
| Proteobacteria;Alphaproteobacteria;Rhizobiales;Methylobacteriaceae | 1.13 | 0.18 | 0.04 |
| Proteobacteria;Alphaproteobacteria;Rhodospirillales;Other | 1.13 | 0.18 | 0.09 |
| Actinobacteria;Actinobacteria;Actinomycetales;Micromonosporaceae | 1.11 | 0.48 | 0.19 |
| Actinobacteria;Actinobacteria;Actinomycetales;Nocardioidaceae | 1.04 | 0.31 | 0.08 |
| Proteobacteria;Gammaproteobacteria;Pseudomonadales;Moraxellaceae | 1.03 | 0.22 | 0.10 |
| Proteobacteria;Betaproteobacteria;Burkholderiales;Burkholderiales_incertae_sedis | 1.03 | 0.51 | 0.22 |
| Proteobacteria;Alphaproteobacteria;Rhizobiales;Phyllobacteriaceae | 1.02 | 0.11 | 0.03 |
| Actinobacteria;Actinobacteria;Actinomycetales;Microbacteriaceae | 1.01 | 0.18 | 0.07 |
| OD1;OD1_genera_incertae_sedis;Other;Other | 1.01 | 0.05 | <0.01 |
| Actinobacteria;Actinobacteria;Acidimicrobiales;Iamiaceae | 1 | 0.30 | 0.17 |
| Actinobacteria;Actinobacteria;Actinomycetales;Streptomycetaceae | 0.99 | 0.35 | 0.20 |
| Acidobacteria;Acidobacteria_Gp6;Gp6;Other | 0.93 | 2.34 | 1.36 |
| Firmicutes;Bacilli;Bacillales;Bacillaceae | 0.92 | 2.02 | 1.21 |
| Proteobacteria;Alphaproteobacteria;Rhizobiales;Rhizobiaceae | 0.91 | 0.06 | 0.03 |
| Proteobacteria;Alphaproteobacteria;Caulobacterales;Caulobacteraceae | 0.91 | 0.47 | 0.29 |
| Bacteroidetes;Flavobacteria;Flavobacteriales;Flavobacteriaceae | 0.9 | 0.06 | <0.01 |
| Proteobacteria;Deltaproteobacteria;Syntrophobacterales;Syntrophobacteraceae | 0.89 | <0.01 | 0.15 |
| Bacteroidetes;Sphingobacteria;Sphingobacteriales;Sphingobacteriaceae | 0.88 | 0.15 | 0.08 |
| Firmicutes;Bacilli;Bacillales;Paenibacillaceae | 0.88 | 0.15 | 0.07 |
| Actinobacteria;Actinobacteria;Acidimicrobidae_incertae_sedis;Ilumatobacter | 0.88 | 0.01 | <0.01 |
| Actinobacteria;Actinobacteria;Solirubrobacterales;Conexibacteraceae | 0.88 | 0.05 | 0.01 |
| Proteobacteria;Alphaproteobacteria;Sphingomonadales;Other | 0.85 | 0.07 | 0.02 |
| Proteobacteria;Betaproteobacteria;Burkholderiales;Comamonadaceae | 0.85 | 0.31 | 0.22 |
| Actinobacteria;Actinobacteria;Actinomycetales;Micrococcaceae | 0.84 | 0.03 | <0.01 |
| Proteobacteria;Gammaproteobacteria;Xanthomonadales;Xanthomonadaceae | 0.84 | 0.86 | 0.62 |
| Unclassified;Other;Other;Other;Other | 0.83 | 0.10 | 0.06 |
| Verrucomicrobia;Verrucomicrobiae;Verrucomicrobiales;Verrucomicrobiaceae | 0.82 | 0.04 | 0.01 |
| Firmicutes;Bacilli;Bacillales;Other | 0.81 | 0.39 | 0.23 |
| Proteobacteria;Deltaproteobacteria;Other;Other | 0.78 | 0.85 | 0.72 |
| Proteobacteria;Gammaproteobacteria;Xanthomonadales;Sinobacteraceae | 0.78 | 0.07 | 0.01 |
| Proteobacteria;Deltaproteobacteria;Myxococcales;Other | 0.78 | 1.57 | 1.42 |
| Proteobacteria;Betaproteobacteria;Burkholderiales;Other | 0.75 | 0.67 | 0.90 |
| Verrucomicrobia;Other;Other;Other | 0.74 | 0.32 | 0.22 |
| Proteobacteria;Alphaproteobacteria;Rhodospirillales;Acetobacteraceae | 0.74 | 1.68 | 1.44 |
| Proteobacteria;Deltaproteobacteria;Desulfuromonadales;Other | 0.74 | <0.01 | 0.11 |
| Verrucomicrobia;Opitutae;Opitutales;Opitutaceae | 0.73 | 0.19 | 0.11 |
| Actinobacteria;Actinobacteria;Other;Other | 0.73 | 1.25 | 1.02 |
| Actinobacteria;Actinobacteria;Solirubrobacterales;Other | 0.73 | 2.42 | 2.14 |
| Actinobacteria;Actinobacteria;Actinomycetales;Other | 0.7 | 2.74 | 2.59 |
| Other;Other;Other;Other | 0.7 | 19.92 | 18.85 |
| Planctomycetes;Planctomycetacia;Planctomycetales;Planctomycetaceae | 0.7 | 1.20 | 1.24 |
| Firmicutes;Clostridia;Clostridiales;Other | 0.69 | <0.01 | 0.10 |
| Acidobacteria;Acidobacteria_Gp10;Gp10;Other | 0.67 | 0.07 | 0.03 |
| Proteobacteria;Alphaproteobacteria;Rhizobiales;Bradyrhizobiaceae | 0.67 | 4.06 | 4.08 |
| Actinobacteria;Actinobacteria;Actinomycetales;Nocardiaceae | 0.66 | 0.02 | 0.02 |
| Proteobacteria;Alphaproteobacteria;Other;Other | 0.66 | 3.44 | 3.63 |
| Proteobacteria;Other;Other;Other | 0.65 | 2.61 | 2.58 |
| Proteobacteria;Deltaproteobacteria;Myxococcales;Polyangiaceae | 0.64 | 0.85 | 0.70 |
| Actinobacteria;Actinobacteria;Actinomycetales;Nakamurellaceae | 0.63 | 0.06 | 0.04 |
| TM7;TM7_genera_incertae_sedis;Other;Other | 0.63 | 0.94 | 1.05 |
| Acidobacteria;Acidobacteria_Gp7;Gp7;Other | 0.63 | 0.48 | 0.55 |
| Actinobacteria;Actinobacteria;Actinomycetales;Catenulisporaceae | 0.6 | 0.06 | 0.05 |
| Proteobacteria;Betaproteobacteria;Other;Other | 0.6 | 1.72 | 1.80 |
| Acidobacteria;Acidobacteria_Gp16;Gp16;Other | 0.59 | 0.38 | 0.40 |
| Proteobacteria;Deltaproteobacteria;Bdellovibrionales;Bdellovibrionaceae | 0.59 | 0.03 | <0.01 |
| Proteobacteria;Betaproteobacteria;Burkholderiales;Burkholderiaceae | 0.58 | 0.31 | 0.55 |
| Proteobacteria;Alphaproteobacteria;Rhizobiales;Other | 0.57 | 4.72 | 6.79 |
| Actinobacteria;Actinobacteria;Actinomycetales;Kineosporiaceae | 0.56 | 0.04 | 0.01 |
| **Closest bacterial relative** | **Contribution to**  **dissimilarity** | **Proportion of**  **all sequences** | |
|  |  | **Natural**  **forest** | **Natural grassland** |
|  | **%** | | |
| Actinobacteria;Actinobacteria;Actinomycetales;Propionibacteriaceae | 2.31 | 0.65 | 0.01 |
| Acidobacteria;Acidobacteria_Gp4;Gp4;Other | 2.12 | 3.65 | 0.44 |
| Bacteroidetes;Flavobacteria;Flavobacteriales;Flavobacteriaceae | 2.11 | 0.39 | <0.01 |
| Nitrospira;Nitrospira;Nitrospirales;Nitrospiraceae | 2.08 | 1.81 | 0.16 |
| Actinobacteria;Actinobacteria;Solirubrobacterales;Solirubrobacteraceae | 1.9 | 0.24 | 0.02 |
| Proteobacteria;Gammaproteobacteria;Xanthomonadales;Sinobacteraceae | 1.81 | 0.33 | 0.01 |
| Bacteroidetes;Sphingobacteria;Sphingobacteriales;Other | 1.61 | 0.37 | 0.02 |
| Acidobacteria;Acidobacteria_Gp25;Gp25;Other | 1.58 | 0.16 | <0.01 |
| Acidobacteria;Acidobacteria_Gp6;Gp6;Other | 1.5 | 6.39 | 1.36 |
| Actinobacteria;Actinobacteria;Acidimicrobidae_incertae_sedis;Ilumatobacter | 1.46 | 0.06 | <0.01 |
| Acidobacteria;Acidobacteria_Gp11;Gp11;Other | 1.39 | 0.12 | <0.01 |
| Verrucomicrobia;Spartobacteria;Spartobacteria_genera_incertae_sedis;Other | 1.38 | 2.40 | 0.74 |
| Acidobacteria;Acidobacteria_Gp5;Gp5;Other | 1.38 | 1.12 | 0.28 |
| Proteobacteria;Gammaproteobacteria;Pseudomonadales;Pseudomonadaceae | 1.36 | 0.13 | 0.01 |
| Proteobacteria;Alphaproteobacteria;Rhizobiales;Phyllobacteriaceae | 1.35 | 0.20 | 0.03 |
| Bacteroidetes;Sphingobacteria;Sphingobacteriales;Chitinophagaceae | 1.29 | 3.26 | 1.09 |
| Proteobacteria;Deltaproteobacteria;Desulfuromonadales;Geobacteraceae | 1.27 | 0.03 | 0.75 |
| Acidobacteria;Acidobacteria_Gp17;Gp17;Other | 1.26 | 0.17 | 0.03 |
| Acidobacteria;Acidobacteria_Gp22;Gp22;Other | 1.26 | 0.08 | <0.01 |
| Proteobacteria;Deltaproteobacteria;Other;Other | 1.23 | 2.20 | 0.72 |
| Actinobacteria;Actinobacteria;Acidimicrobiales;Iamiaceae | 1.13 | 0.43 | 0.17 |
| Actinobacteria;Actinobacteria;Actinomycetales;Nocardioidaceae | 1.1 | 0.23 | 0.08 |
| Proteobacteria;Gammaproteobacteria;Enterobacteriales;Enterobacteriaceae | 1.09 | 0.08 | <0.01 |
| Chloroflexi;Anaerolineae;Anaerolineales;Anaerolineaceae | 1.08 | 0.03 | 0.78 |
| Actinobacteria;Actinobacteria;Actinomycetales;Micromonosporaceae | 1.07 | 0.45 | 0.19 |
| Bacteroidetes;Sphingobacteria;Sphingobacteriales;Cytophagaceae | 1.05 | 0.09 | 0.02 |
| Verrucomicrobia;Verrucomicrobiae;Verrucomicrobiales;Verrucomicrobiaceae | 1 | 0.07 | 0.01 |
| Actinobacteria;Actinobacteria;Actinomycetales;Pseudonocardiaceae | 0.99 | 0.03 | 0.01 |
| Proteobacteria;Betaproteobacteria;Burkholderiales;Other | 0.99 | 1.21 | 0.90 |
| Proteobacteria;Alphaproteobacteria;Rhodospirillales;Other | 0.99 | 0.22 | 0.09 |
| Proteobacteria;Alphaproteobacteria;Rhizobiales;Other | 0.98 | 10.54 | 6.79 |
| Proteobacteria;Deltaproteobacteria;Syntrophobacterales;Syntrophobacteraceae | 0.97 | <0.01 | 0.15 |
| Acidobacteria;Acidobacteria_Gp7;Gp7;Other | 0.97 | 1.05 | 0.55 |
| Gemmatimonadetes;Gemmatimonadetes;Gemmatimonadales;Gemmatimonadaceae | 0.93 | 0.98 | 0.65 |
| Proteobacteria;Alphaproteobacteria;Rhizobiales;Rhizobiaceae | 0.92 | 0.07 | 0.03 |
| Acidobacteria;Acidobacteria_Gp16;Gp16;Other | 0.92 | 0.70 | 0.40 |
| Acidobacteria;Acidobacteria_Gp15;Gp15;Other | 0.91 | 0.09 | 0.03 |
| Actinobacteria;Actinobacteria;Other;Other | 0.9 | 1.96 | 1.02 |
| Acidobacteria;Acidobacteria_Gp10;Gp10;Other | 0.86 | 0.10 | 0.03 |
| Proteobacteria;Alphaproteobacteria;Rhizobiales;Methylocystaceae | 0.86 | 0.01 | 0.25 |
| Actinobacteria;Actinobacteria;Actinomycetales;Intrasporangiaceae | 0.85 | 0.12 | 0.06 |
| Actinobacteria;Actinobacteria;Solirubrobacterales;Conexibacteraceae | 0.83 | 0.06 | 0.01 |
| Proteobacteria;Gammaproteobacteria;Xanthomonadales;Xanthomonadaceae | 0.83 | 0.91 | 0.62 |
| Bacteria_incertae_sedis;Ktedonobacteria;Ktedonobacterales;Ktedonobacteraceae | 0.82 | 0.10 | 1.17 |
| Proteobacteria;Deltaproteobacteria;Desulfuromonadales;Other | 0.81 | <0.01 | 0.11 |
| Proteobacteria;Alphaproteobacteria;Sphingomonadales;Sphingomonadaceae | 0.78 | 1.66 | 1.42 |
| Proteobacteria;Deltaproteobacteria;Myxococcales;Polyangiaceae | 0.78 | 1.12 | 0.70 |
| Bacteroidetes;Other;Other;Other | 0.77 | 0.70 | 0.47 |
| Proteobacteria;Betaproteobacteria;Burkholderiales;Comamonadaceae | 0.76 | 0.24 | 0.22 |
| Proteobacteria;Gammaproteobacteria;Pseudomonadales;Moraxellaceae | 0.75 | 0.12 | 0.10 |
| Actinobacteria;Actinobacteria;Solirubrobacterales;Other | 0.73 | 2.81 | 2.14 |
| Actinobacteria;Actinobacteria;Actinomycetales;Streptomycetaceae | 0.72 | 0.23 | 0.20 |
| Unclassified;Other;Other;Other;Other | 0.7 | 0.04 | 0.06 |
| Proteobacteria;Other;Other;Other | 0.69 | 2.55 | 2.58 |
| OD1;OD1_genera_incertae_sedis;Other;Other | 0.69 | 0.02 | <0.01 |
| Proteobacteria;Alphaproteobacteria;Rhizobiales;Hyphomicrobiaceae | 0.69 | 1.65 | 0.52 |
| Firmicutes;Clostridia;Clostridiales;Veillonellaceae | 0.68 | <0.01 | 0.07 |
| Verrucomicrobia;Other;Other;Other | 0.68 | 0.24 | 0.22 |
| Firmicutes;Bacilli;Bacillales;Planococcaceae | 0.67 | 0.04 | 0.01 |
| OP10;OP10_genera_incertae_sedis;Other;Other | 0.67 | 0.01 | 0.17 |
| Firmicutes;Clostridia;Clostridiales;Clostridiaceae | 0.64 | <0.01 | 0.06 |
| Other;Other;Other;Other | 0.63 | 19.72 | 18.85 |
| Chloroflexi;Thermomicrobia;Other;Other | 0.63 | <0.01 | <0.01 |
| Acidobacteria;Acidobacteria_Gp13;Gp13;Other | 0.61 | 0.04 | 0.03 |
| Proteobacteria;Deltaproteobacteria;Syntrophobacterales;Syntrophaceae | 0.6 | <0.01 | 0.05 |
| Proteobacteria;Gammaproteobacteria;Methylococcales;Methylococcaceae | 0.59 | <0.01 | 0.08 |
